# Immune response against SARS-CoV-2 variants: the role of neutralization assays

**DOI:** 10.1038/s41541-021-00404-6

**Published:** 2021-11-29

**Authors:** Alicja Maria Chmielewska, Anna Czarnota, Krystyna Bieńkowska-Szewczyk, Katarzyna Grzyb

**Affiliations:** Laboratory of Virus Molecular Biology, Intercollegiate Faculty of Biotechnology, University of Gdańsk, Gdańsk, Poland

**Keywords:** Viral infection, Viral infection

## Abstract

Since the emergence of the novel coronavirus SARS-CoV-2 in late 2019, the COVID-19 pandemic has hindered social life and global economic activity. As of July 2021, SARS-CoV-2 has caused over four million deaths. The rapid spread and high mortality of the disease demanded the international scientific community to develop effective vaccines in a matter of months. However, unease about vaccine efficacy has arisen with the spread of the SARS-CoV-2 variants of concern (VOCs). Time- and cost-efficient in vitro neutralization assays are widely used to measure neutralizing antibody responses against VOCs. However, the extent to which in vitro neutralization reflects protection from infection remains unclear. Here, we describe common neutralization assays based on infectious and pseudotyped viruses and evaluate their role in testing neutralizing responses against new SARS-CoV-2 variants. Additionally, we briefly review the recent findings on the immune response elicited by available vaccines against major SARS-CoV-2 variants, including Alpha, Beta, Gamma, and Delta.

## Introduction

The outbreak of coronavirus disease 2019 (COVID-19) has posed serious threats to global public health systems. COVID-19 is caused by a new single-stranded RNA virus, SARS-CoV-2, which belongs to the *Coronaviridae* family^1^. Since the SARS-CoV-2 outbreak was classified as a pandemic, the development of effective antiviral treatments and vaccines has become the most urgent goal for the scientific community. As of July 2021, four COVID-19 vaccines have been approved for large-scale immunizations in the European Union, and three have been approved for use in the United States^2,3^. All these vaccines use the spike glycoprotein (S protein) as an immunogen because of its essential role in the viral entry process. The S protein contains a receptor-binding domain (RBD) that specifically recognizes the host-cell receptor ACE2; therefore, the RBD represents the main target for neutralizing antibodies elicited during natural infection or after vaccination^4^. The levels of neutralizing antibodies correlate with COVID-19 protection, and data from vaccine clinical trials show that vaccine-elicited neutralizing antibodies are able to effectively prevent the disease^5,6^. However, in September 2020, the first SARS-CoV-2 variant, the Alpha variant, emerged in the United Kingdom, which caused concern due to its enhanced transmissibility and pathogenicity. As of July 2021, three additional variants of concern (VOCs) that carry multiple changes within the S protein have been identified in South Africa (Beta variant), Brazil (Gamma variant), and India (Delta variant)^7^.

In addition to enhanced transmissibility and/or pathogenicity, VOCs might have the ability to evade natural or vaccine-induced immunity. All the currently authorized COVID-19 vaccines are based on the original Wuhan-Hu-1 S protein sequence reported in January 2020, which lacks VOC mutations. With several vaccines being rolled out globally, the important question is whether the emergence of SARS-CoV-2 VOCs might diminish the effectiveness of vaccines or overcome natural immunity, leading to an increased risk of reinfections.

The recent emergence of multiple SARS-CoV-2 variants highlights the importance of the continuous monitoring of variants circulating in the population and the assessment of their sensitivity to neutralization by immune sera. Several in vitro assays have been developed to study the neutralizing activity of the antibodies elicited during infection or after vaccination. Neutralization assays of patient-isolated infectious SARS-CoV-2, recombinant virus, or pseudovirus models are used as convenient tools to analyze the immune responses induced by COVID-19 vaccines against emerging viral variants. Here, we describe the general protocols and functionality of currently available in vitro neutralization tests and discuss their important role in investigating immune responses against new SARS-CoV-2 variants. Additionally, we provide a brief overview of the recent findings on natural and vaccine-induced protection against VOCs.

## Emergence of SARS-CoV-2 variants of concern

The emergence of SARS-CoV-2 variants with several changes in the amino-acid sequence has become an important issue regarding COVID-19 control efforts. Up to July 2021, the WHO has identified four variants of SARS-CoV-2 characterized by higher transmissibility, increased virulence, or ability to escape diagnostics, vaccines, and therapeutics. The variants were defined as variants of concern (VOCs), and include the Alpha variant (formerly B.1.1.7) isolated in September, 2020, in the United Kingdom^8^; Beta variant (formerly B.1.351), isolated in August, 2020, in South Africa^9,10^; Gamma variant (formerly P.1), first described in Brazil and Japanese tourists at the end of 2020^11,12^; and Delta variant (formerly B.1.617.2), first reported in Maharashtra state, in India, in late 2020^13^. As of July 2021, based on sequenced samples deposited to GISAID, the Delta variant is dominant in many countries, including India, the United Kingdom, Israel, Russia, and the United States, and is expected to also become dominant in several other countries.

**Fig. 1:**
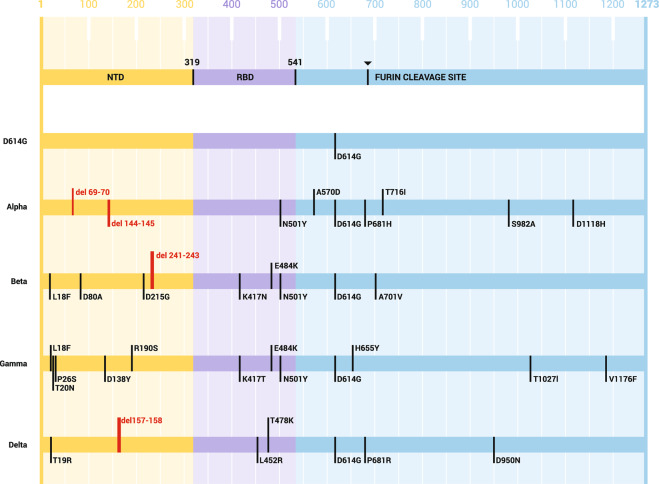
Amino-acid changes in the S protein of the Alpha, Beta, Gamma, and Delta variants of concern of SARS-CoV-2. NTD N-terminal domain, RBD receptor-binding domain.

All four circulating VOCs share a common, non-synonymous mutation, shifting D614 to G614, which was reported in early March 2020. By the end of June 2020, D614G variant became the dominant variant worldwide^14^. The specific sequence changes associated with each of the VOCs are depicted in Fig. 1. The exact mechanism of the emergence of VOCs is unclear. It is possible that the rapid emergence of SARS-CoV-2 variants is influenced by different factors: the high rate at which the virus has spread globally, the infection of immunocompromised individuals allowing prolonged virus circulation in the infected organism, and the use of convalescent plasma therapy with possible suboptimal titers of neutralizing antibodies^15,16^. New SARS-CoV-2 variants emerge constantly; therefore, it is crucial to monitor their spread, virulence, and potential immune evasion.

## Neutralizing antibody assays for SARS-CoV-2

### Infectious SARS-CoV-2 neutralization assays

A high level of neutralizing antibodies against SARS-CoV-2 is recognized as a correlate of protection^17^. Thus, in vitro analysis of virus neutralization by elicited antibodies is a convenient and time-efficient method for monitoring functional antiviral responses against new SARS-CoV-2 variants.

**Fig. 2:**
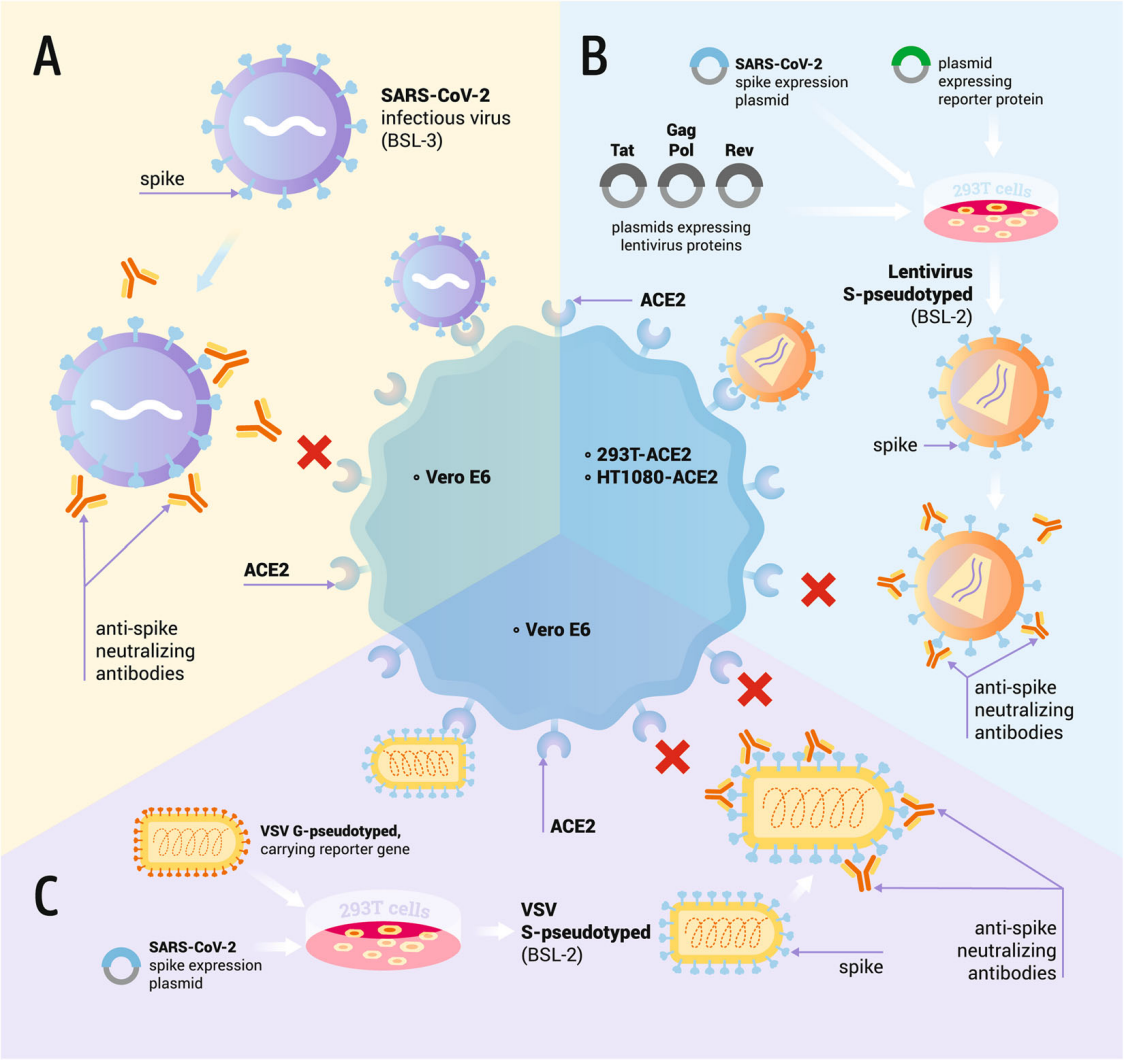
Examples of the approaches used for testing antibody-mediated SARS-CoV-2 neutralization. **A** Cell culture-amplified SARS-CoV-2 infects cells expressing ACE2 receptor, e.g., Vero E6. When incubated with sera, neutralizing antibodies bind to the virus surface and block the interaction between virus spike (S) protein and ACE2 receptor, inhibiting virus entry. **B** 293T cells are transfected with plasmids coding for lentiviral structural and non-structural proteins, and SARS-CoV-2 spike and reporter protein. The produced spike-pseudotyped lentivirus vector enters ACE2-overexpressing cells (e.g., 293-ACE2 or HT1080-ACE2). Spike-ACE2 interaction can be blocked by neutralizing antibodies binding the SARS-CoV-2 spike. Reporter protein expression allows for the measurement of infection rate. **C** 293T cells are transfected with plasmid coding for SARS-CoV-2 spike, followed by infection with G-pseudotyped VSV carrying a reporter gene in the locus of the G-protein sequence. Cells produce spike-pseudotyped VSV particles that can enter cells expressing ACE2, e.g., Vero E6. Pseudovirus with the G gene deleted is only able to carry out a single round of infection. Cell entry can be inhibited by spike-binding SARS-CoV-2 neutralizing antibodies. Reporter protein expression allows for the measurement of infection rate.

Patient-isolated SARS-CoV-2 replicates efficiently in human and non-human cell lines expressing the ACE2 receptor, including Calu-3, Caco-2, Huh.7, LLCMK2, Vero CCL-81, and Vero E6^18,19^. To investigate virus neutralization, immune sera or antibodies are serially diluted and incubated with infectious SARS-CoV-2 to enable antibody binding to viral epitopes (Fig. 2A). Next, the mixture of virus and antibodies is added to permissive cells, most commonly Vero E6. Neutralizing antibodies block the entry of viral particles into the cells, resulting in the suppression of infection, and the rate of neutralization is calculated as the reduction in infectivity with respect to the infection for the non-neutralizing control. The reference neutralization assay is the plaque-reduction neutralization test (PRNT). In this assay, the reduction in the number of viral plaques formed by infectious viral particles in a cell monolayer indicates the extent of virus neutralization^20^. Other conventional assays involve infectivity readout by the immunostaining of viral foci in a cell monolayer or the calculation of the median tissue culture infectious dose (TCID50)^21^. Construction of recombinant SARS-CoV-2 viruses encoding fluorescent or bioluminescent reporter proteins at the ORF7 of the viral genome enabled the rapid analysis of infection rates by automatic infectivity reading, facilitating high-throughput neutralization testing^20,22,23^. Muruato et al. reported comparable neutralization results using reporter SARS-CoV-2 and the conventional plaque-reduction assay, whereas the reporter system reduced the time- and work-load necessary to perform the experiment^20^. Additionally, recombinant SARS-CoV-2 carrying selected mutations or foreign S protein sequences were used as a tool to investigate the neutralization of variants of concern^24–26^. However, it should be mentioned, that viruses isolated from patients comprise a full set of changes not only in the S protein but also in the other structural and non-structural proteins. As the infection is determined not only by virus binding and entry but also by intracellular virus propagation and exit pathways, in vitro models using patient isolates are most likely to provide the most relevant neutralization results.

### Pseudovirus-based neutralization tests

Since neutralization studies using replication-competent SARS-CoV-2 require biosafety level 3 (BSL3) containment, they are limited to specialized, access-restricted facilities. To avoid this problem, envelope proteins of highly pathogenic viruses can be presented on the backbones of other viruses, e.g., retroviruses. HIV-1 and VSV pseudotyped with the SARS-CoV-2 S protein have been successfully used for studies of COVID-19 vaccine-induced entry and neutralization^27,28^.

#### Lentivirus-based pseudotype viruses

Pseudotyped viruses based on retro- and lentiviruses have been used as a surrogate platform to study numerous high-containment pathogens or viruses that do not easily propagate in cell culture^29^. Before the development of a cell-culture system for hepatitis C virus (HCV), retroviruses pseudotyped with HCV surface glycoproteins were the first in vitro infection system that enabled the functional investigation of the entry and neutralization of this pathogen^30^.

Recently, pseudotyped viruses based on HIV-1 have been successfully applied for studies of SARS-CoV-2 (Fig. 2B). HIV-1 is a hazard-group-3 pathogen, but as HIV-1-based pseudotyped viruses are not replication-competent, they can be handled in BSL2 facilities^29^.

Thorough protocols describing the production and neutralization of SARS-CoV-2 S-pseudotyped lentivirus are available^31,32^. Generally, lentiviral particles are generated by the co-transfection of HEK293T cells with a set of plasmids carrying HIV-1 structural and non-structural genes, a reporter gene (e.g., GFP or luciferase) flanked by HIV long terminal repeats (LTRs), and a plasmid expressing the SARS-CoV-2 S protein. The reporter gene sequence is packed into lentiviral capsids assembled by cellular machinery, and the S protein is embedded in the lentiviral envelope. To assess the rate of neutralization, pseudotyped viruses are incubated with serially diluted antibodies and added to target cells. 293T and HT1080 cells overexpressing the ACE2 receptor and Vero cells overexpressing ACE2 and the spike-activating protease (TPMRSS2) have been reported as permissive for S-pseudotyped lentivirus, whereas cells expressing natural levels of ACE2 cannot be infected efficiently. After infection, the rate of neutralization is reflected by the reduction in the reporter protein expression in pseudovirus-infected cells^31,32^.

#### VSV-based pseudotyped viruses

Vesicular stomatitis virus (VSV) is a negative-sense RNA rhabdovirus primarily infecting animals (e.g., cattle). The bullet-shaped nucleocapsid is surrounded by a lipid envelope with the surface glycoprotein G, which mediates the virus entry. Glycoprotein G-deleted VSV pseudotyped with heterologous surface glycoproteins has been used extensively both as a molecular tool for entry studies and as a vaccine-vector candidate. In 2019, chimeric VSV carrying the Zaire Ebola virus glycoprotein was approved as the first vector-based human vaccine. VSV pseudovirus studies can be performed in BSL2 facilities, making it a convenient model for high-containment pathogens^33^.

Nie et al.^34^, Almahboub et al.^35^, and Capcha et al.^36^ described detailed protocols for the generation and neutralization of SARS-CoV-2 S protein-pseudotyped VSV. Briefly, 293T or BHK-21 cells are transfected with a plasmid coding for the SARS-CoV-2 S protein (Fig. 2C). The transfected cells are infected with VSV pseudotyped with the VSV glycoprotein G. This virus contains a defective genome with a reporter gene inserted in the place of the G-protein sequence, however, it still bears G protein on its surfaces, which is produced from a helper plasmid and acquired from the cell membrane. After cell infection, the defective VSV genome replicates and provides a basis for the production of all VSV proteins, except for protein G. The assembled viral particles bud from the cell, acquiring envelope together with cell membrane-bound S proteins. S protein-pseudotyped VSV generally enters ACE2-expressing cells that are naturally permissive to SARS-CoV-2, e.g., Vero E6, Vero CCL-81, Calu-3, Caco-2, and Huh.7. As in target cells no surface protein is produced, a new generation of infectious virus particles is not formed. Thus, the pseudovirus with a deleted G gene is only able to carry out a single round of infection.

VSV can also be engineered as a chimeric, replication-competent virus carrying the SARS-CoV-2 S protein in its envelope^37,38^. Replicating VSV contains the S protein gene in place of the native G-protein sequence. Chimeric VSV amplifies efficiently in cell culture and mutates at a high rate, which makes it useful for virus evolution and immune escape studies^15^.

VSV and lentiviral pseudoviruses do not require enhanced biosafety measures and are efficiently produced in cell culture. A pseudovirus’ infectivity can be further enhanced by modifications to the S protein, including C-terminal 19 amino-acid truncation or the overexpression of host factors in target cells, e.g., the spike-activating protease TMPRSS2^39,40^. Once the new S protein sequence is known, pseudotyped virus can be generated more easily and quickly than the whole infectious virus. Furthermore, pseudotyped virus is a perfect tool to investigate single mutations that can be easily introduced into S protein-coding plasmid vector using standard genetic engineering methods. Together with the simplicity of reporter systems this makes pseudotyped viruses a potent tool for monitoring immune responses against numerous SARS-CoV-2 variants. However, it should be noted that pseudovirus-based assays only detect neutralizing antibodies that bind to S protein epitopes and cannot detect potential antibodies binding to other SARS-CoV-2 proteins. Several comparative studies have analyzed pseudovirus neutralization in reference to replication-competent SARS-CoV-2. They showed robustness, high repeatability, and good correlation with conventional replication-competent virus-neutralization titers^21,37^.

### Antibody-binding assays

Serological assays that measure the levels of antibodies binding the SARS-CoV-2 S protein are extremely useful for serodiagnosis. However, because they do not differentiate between neutralizing antibodies and S protein-binding non-neutralizing antibodies, they are not suitable for evaluating SARS-CoV-2 neutralization-mediated protection. Conversely, competitive serological assays detect functional antibodies that block the interaction between the SARS-CoV-2 receptor-binding domain (RBD) and recombinant ACE2 receptor, and so can be used as a screening tool to evaluate potential neutralization^41^. As such, these assays do not require the use of cell cultures and are easy to standardize and perform. However, there are reports of neutralizing antibodies binding to epitopes other than those in the RBD that cannot be detected in RDB–ACE2 competitive assays^42^.

## Vaccine-induced and natural immunity against VOCs: what we know so far

### Neutralization of VOCs by convalescent sera

One important concern regarding the emergence of SARS-CoV-2 variants is whether SARS-CoV-2 immunity acquired during the previous infection is protective against VOCs. Nearly all research regarding SARS-CoV-2 variants neutralization by convalescent sera has been performed using in vitro neutralization assays. Studies of Alpha variant have demonstrated that convalescent plasma can fully cross-neutralize the infectious virus and only 1.5–2.5-fold decreased neutralization was observed for a pseudovirus or recombinant SARS-CoV-2 carrying Alpha variant S protein in comparison with a non-VOC virus^26,43,44^. This suggests that previous infection with wild-type SARS-CoV-2 still provides protection against the Alpha variant.

On the other hand, several studies have reported that Beta and Gamma variants can evade neutralization by the sera from patients previously infected with non-VOC virus. Both Beta and Gamma variants carry identical RBD changes, and assays performed using pseudoviruses or infectious viruses carrying those changes showed significant reductions in neutralization by convalescent plasma^27,45–47^. Comprehensive studies by Wang et al. showed that for Beta and Gamma variant isolates, neutralization was reduced 9.4- and 3.4-fold, respectively, and even greater reductions—22 and 6.5-fold—were observed for the VSV-based pseudoviruses carrying whole sets of Beta and Gamma S protein changes, respectively^48,49^. Similarly, preliminary data on the Delta variant suggest decreased neutralization by convalescent plasma. A recent study by Planas et al. reported that for the Delta variant, the neutralization titers were significantly decreased by 4-fold to 6-fold in comparison to the isolates from Alpha and wild-type strains, respectively^50^. Interestingly, in this study, reductions in neutralizing titers were comparable for Beta and Delta VOCs.

The only available real-world data on the effect of naturally acquired SARS-CoV-2 immunity on VOC infection derive from observations in Manaus, Brazil. Widespread transmission of the Gamma variant has been reported in this region, despite seroprevalence against non-VOC SARS-CoV-2 above the theoretical herd immunity threshold, raising concern that infection with non-VOCs may not fully protect from reinfection^12,51^. Although this could be explained by the waning immunity or overestimation of seroprevalence in the first place, significant reductions in the neutralization titers reported for Beta, Gamma, and Delta VOCs suggest the risk of reinfection of convalescent individuals, especially those with a weak or waning humoral response.

### Vaccine-induced immunity against VOCs

#### Immunity induced by the mRNA-based vaccines

The BNT162b2 vaccine from Pfizer and the mRNA-1273 vaccine from Moderna are based on lipid nanoparticle-encapsulated mRNA, coding for S protein derived from SARS-CoV-2 isolated in January 2020, which therefore lacks VOC mutations. The efficacy of the vaccines was determined in clinical trials that started in late spring, 2020. Both BNT162b2 and mRNA-1273 demonstrated efficacy greater than 90% in preventing symptomatic COVID-19.

Currently, in vitro neutralization is the method of choice for the high-throughput assessment of mRNA-based vaccines’ ability to elicit neutralizing antibodies against VOCs. In numerous studies, no or only a slight reduction in neutralizing titers was reported for pseudotyped or recombinant viruses carrying the Alpha variant S protein (Table 1). This suggests that both BNT162b2 and mRNA-1273 vaccines should offer protection against this variant. For the neutralization assay with constructs carrying the S protein derived from Beta or Gamma isolates, significantly decreased neutralization titers were observed, suggesting the possibility of reduced BNT162b2 and mRNA-1273 vaccine effectiveness against those VOCs. Although limited data are available for the Delta VOC, studies have shown moderate to strong reductions in neutralization titers elicited by the mRNA-based vaccines. The results from selected studies are summarized in Table 1.

**Table 1:** Review of neutralization studies with sera elicited by mRNA-based vaccines.

Vaccine	Neutralization system	Changes in S protein	Reduction in neutralization titers (fold)	Refs.
BNT162b2	Lentivirus-based pseudovirus	N501Y	None	^75^
BNT162b2	Recombinant SARS-CoV-2	N501Y	None	^24^
BNT162b2	Recombinant SARS-CoV-2	Selected – Alpha	None	^25^
BNT162b2	Isolate	Alpha	2	^26^
BNT162b2	Isolate	Alpha	2.4	^73^
BNT162b2	Isolate	Alpha	3.3	^76^
BNT162b2	Isolate	Alpha	None	^48^
BNT162b2	Isolate	Alpha	2.6	^77^
mRNA-1273	Isolate	Alpha	None	^48^
mRNA-1273	Lentivirus-based pseudovirus	Alpha	2.1	^43^
BNT162b2	VSV-based pseudovirus	Alpha	None	^78^
mRNA-1273	VSV-based pseudovirus	Alpha	None	^28^
BNT162b2	Isolate	Beta	4.5	^73^
BNT162b2	Isolate	Beta	10.3	^48^
BNT162b2	Isolate	Beta	7.6	^79^
BNT162b2	Isolate	Beta	16 (compared to Alpha)	^50^
BNT162b2	Isolate	Beta	4.9	^77^
mRNA-1273	Isolate	Beta	12.4	^48^
mRNA-1273	Isolate	Beta	3.8	^80^
BNT162b2	Recombinant SARS-CoV-2	Beta	9	^26^
mRNA-1273	VSV-based pseudovirus	Beta	6.4	^28^
BNT162b2	Lentivirus-based pseudovirus	Beta, Gamma RBD	6.8	^75^
BNT162b2 and mRNA-1273	Lentivirus-based pseudovirus	Beta, Gamma RBD	1–3	^27^
BNT162b2	Recombinant SARS-CoV-2	Beta, Gamma RBD	1.3	^25^
BNT162b2	Isolate	Gamma	3.8	^49^
mRNA-1273	Isolate	Gamma	4.8	^49^
BNT162b2	VSV-based pseudovirus	Gamma	2.2	^49^
mRNA-1273	VSV-based pseudovirus	Gamma	2.8	^49^
BNT162b2	Isolate	Delta	3 (compared to Alpha)	^50^
BNT162b2	Isolate	Delta	5.8	^77^

The reduction in neutralization observed for Beta, Gamma or Delta variants could potentially have serious ramifications for the roll-out of mRNA vaccines in regions impacted by those VOCs. Therefore, vaccine companies are working on booster doses of vaccines designed to protect from COVID-19 caused by VOCs. Moderna have published results from a booster vaccination trial with Beta variant-matched mRNA (mRNA-1273.351) in previously vaccinated individuals, where neutralization titers against Beta and Gamma VOC pseudoviruses were comparable with the titers against wild-type virus elicited by the mRNA-1273 vaccination^52^.

Neutralization results cannot be simply translated into vaccine effectiveness in the field. Some evidence for the reduced effectiveness of mRNA-based vaccines against VOCs has recently been provided. In a study from Israel, Kustin et al.^53^ suggested that individuals fully vaccinated with BNT162b2 might be more likely to become infected with the Beta VOC. However, the study was limited by the small sample size. A study carried out in Qatar^54^ demonstrated a reduction in BNT162b2 vaccine effectiveness against infection with Alpha and Beta VOCs—with 89.5% and 75% effectiveness, respectively. However, BNT162b2 still provided protection from severe COVID-19 with 97.4% effectiveness. In a recent study by Sheikh et al.^55^, BNT162b2 vaccine effectiveness against infection was shown to drop from 92% for the Alpha variant to 79% for the Delta variant; similar results were reported by Bernal et al.^56^, showing that in fully vaccinated individuals, BNT162b2 vaccine effectiveness against symptomatic COVID-19 was reduced from 93.4% to 87.9%. Most importantly, a study from Stowe et al. by Public Health England showed that although BNT162b2 effectiveness against infection was reduced, the effectiveness of the vaccine against hospitalization was similar for both Alpha and Delta variants, with 95% and 96%, respectively^57^.

#### Adenovirus-based vaccine-induced immunity

AstraZeneca’s AZD1222 vaccine is based on a replication-deficient chimpanzee adenoviral vector carrying the sequence coding for SARS-CoV-2 S protein^58^. The Johnson & Johnson Ad26.COV2.S vaccine is based on a replication-deficient human adenovirus type 26 vector, coding for the S protein in the stabilized prefusion conformation^59^. Both Johnson & Johnson and AstraZeneca were able to design trials to test the efficacy of their vaccines against Alpha and Beta VOCs during phase III clinical trials. Interestingly, this allowed for direct comparisons of the clinical efficacy of the vaccines against VOCs with neutralization data. AZD1222 attained an efficacy against symptomatic infection of 81.5% for the non-VOC isolate, which was reduced to 70.4% for the Alpha VOC^60^, with confidence intervals overlapping to a large extent. Interestingly, in vitro neutralization exhibited 9-fold reduced titers for the Alpha variant in comparison with the non-VOC^60^. Those results revealed substantial disparities between the reduction in neutralization titers in vitro and clinical vaccine efficacy. In further studies, AZD1222 appeared to provide only minimal protection against mild to moderate COVID-19 caused by the Beta variant in a South African cohort—the final vaccine efficacy reached only 10.6% with a 92% Beta variant prevalence in the sequenced samples derived from symptomatic participants^61^. Accordingly, in vitro neutralization studies showed significantly reduced titers against the Beta isolate^61^. The vaccine’s efficacy against severe COVID-19 was not determined. Because of the reduced efficacy of AZD1222 against Beta strain, a new vaccine, called AZD2816, which carries Beta variant S protein sequence, will be administered to AZD1222 recipients and non-vaccinated individuals in clinical trials, in the hope of eliciting antibodies neutralizing the Beta VOC. Recently tested neutralizing titers elicited by AZD122 showed a 5-fold reduction against Delta relative to Alpha^50^. However, Bernal et al. showed that in fully vaccinated individuals, AZD122 vaccine effectiveness was reduced only from 66.1% to 59.8% for Alpha and Delta variants, respectively^56^. Moreover, the effectiveness of AZD1222 against hospitalization was significantly higher: 86% against the Alpha variant and 92% against the Delta variant^57^.

Johnson & Johnson announced that the efficacy of their Ad26.COV2.S vaccine was 66% against moderate to severe COVID-19 across all study locations^59^. Specifically, in South Africa, with a 95% Beta variant prevalence in the sequenced samples, the efficacy against the severe disease was 85%, and that against moderate to severe disease was 57%^59^. The efficacy data did not include individuals with mild COVID-19, which probably resulted in a higher reported vaccine efficacy in comparison to AZD1222. A recently published study involving sera from individuals immunized using Ad26.COV2.S vaccine showed no reduction in the neutralization titer for pseudotyped lentivirus carrying S protein changes derived from the Alpha variant, reduced neutralization for a pseudovirus carrying Beta (3.6-fold) and Gamma (3.4-fold) variant S protein, and slight reductions for a pseudovirus carrying the Delta variant S protein (1.5-fold)^62^.

## Standardization of neutralization assays

In vitro neutralization tests are based on a variety of protocols, using several infectious agents, cell lines, and reporter systems. This makes direct comparisons of results from separate studies extremely difficult. With the aim of minimizing the inter-laboratory variation of anti-SARS-CoV-2 antibody assays, the Coalition for Epidemic Preparedness Innovations (CEPI), the National Institute for Biological Standards and Control (NIBSC), and the WHO have established standardized reagents consisting of pooled COVID-19 convalescent plasma samples. They are available as the International Standard and the International Reference Panel for Anti-SARS-CoV-2 Immunoglobulin at NIBSC^63,64^. Standardized plasma samples are meant to be used for the calibration of assays to an arbitrary international unit (IU) in neutralization assays, and a binding antibody unit (BAU) in binding assays. The scientific community is strongly encouraged to report the data of antibody assays relative to the International Standard units to enable straightforward comparisons of antibody neutralizing and binding titers^65^.

Furthermore, in October 2020, CEPI set up a network of laboratories to assess vaccine immunogenicity using standardized reagents and protocols. The network provides free-of-charge analyses of cellular and humoral responses induced by vaccines in pre-clinical and clinical trials, including the neutralization of wild-type SARS-CoV-2 and pseudoviruses^66^. From July 2021, the network has started evaluations of candidate and rolled-out vaccines against VOCs. Data provided by the network will be extremely valuable for direct comparisons of different vaccination approaches against SARS-CoV-2 variants^67^.

In addition to the standardization of assays, a key challenge in the field is to define the relationship between immune responses measured in vitro and COVID-19 protection. Several recent studies have analyzed data from clinical trials to establish correlates of protection for SARS-CoV-2 infection. Earle et al. demonstrated a robust correlation between neutralizing and binding antibody titers and the efficacy of seven different vaccines in phase III clinical trials^68^. Khoury et al. provided a quantitative prediction of the correlation between neutralizing antibody titers and clinical protection, based on the data from vaccinated and convalescent cohorts. The presented model includes analysis of protection against SARS-CoV-2 variants and predicts that a reduction in the neutralization titer against VOCs will have a greater impact on vaccines with lower baseline efficacy against the non-VOC virus^6^. However, limitations of both studies are the high rates of variability in clinical protocols, vaccine platforms, population samples, study locations, and protocols of serological assays. Despite these constraints, both studies support the use of antibody assays as predictors of vaccine efficacy.

## Conclusions

The major concern regarding the control of the COVID-19 pandemic is an emergence and rapid spread of new SARS-CoV-2 variants that could overcome acquired immune protection. Clinical evaluations of candidate and rolled-out vaccines against the emerging SARS-CoV-2 variants are time-consuming, therefore, there is a need for fast and straightforward testing. Most data on immune responses against the SARS-CoV-2 variants have been collected using in vitro neutralization tests, which are fast to perform and allow for rapid assay adjustment by inserting new variant sequences or single mutations into vectors using genetic engineering methods. Therefore, they are an ideal tool for screening numerous virus variants as they occur in the population. Moreover, because effective COVID-19 vaccines are available worldwide, the development of new vaccines using placebo-control trials is controversial, and, most likely, will not be possible in future, which will further increase the role of in vitro assays as predictors of vaccine effectiveness.

However, the variety of assay protocols hinders direct comparisons of results from studies, and the relationship between measured neutralization and protection is still unclear. To overcome assay variability, it is postulated that one golden assay based on a specific protocol should be established, which would serve as a reference for in vitro neutralization analysis. Additionally, it is recommended that the results of serological tests should be expressed in international units in reference to the WHO-approved International Standard. Moreover, determining correlates of protection against SARS-CoV-2 variants, including defining precise thresholds of protection, would be invaluable for vaccine studies and could also allow for the assessment of individual risks of symptomatic or severe COVID-19.

It is worth mentioning that, besides the neutralizing activity of anti-SARS-CoV-2 antibodies, other components of the humoral immune response may contribute to infection control, including antibody-dependent cellular cytotoxicity (ADCC) and antibody-dependent complement deposition (ADCD)^69^. Furthermore, T cell responses may contribute to protection from COVID-19, especially in the presence of suboptimal neutralizing antibody titers. Both mRNA- and adenovirus-based vaccines elicit robust anti-S protein T cell responses^70–72^ and recent studies indicate that the response to cellular epitopes is not substantially affected by VOC mutations^73,74^. Therefore, defining immune correlates of protection against COVID-19 should not be limited to antibody neutralization titers, but include antibody binding and cellular response assays.

As several in vitro studies have observed slight to substantial differences in serum neutralizing activity against SARS-CoV-2 variants of concern, clinical observations confirm vaccine effectiveness, especially against moderate/severe disease. However, to avoid a potential loss of effectiveness of COVID-19 vaccines, booster immunizations with vaccines containing VOC immunogens might be necessary. A great advantage of mRNA- and adenovirus vector-based platforms is the possibility of relatively quickly replacing immunogen sequences, while using already optimized delivery platforms. Currently available vaccines could be modified to carry full sequences of newly emerging VOCs or selected mutations determined to confer protection against multiple variants. Additionally, the design of new vaccines targeting the conserved regions of the SARS-CoV-2 S protein could be beneficial in terms of protection against newly arising variants.

## Data Availability

All data generated or analyzed during this study are included in this article.
